# Genome-wide association mapping identifies common bunt (*Tilletia caries*) resistance loci in bread wheat (*Triticum aestivum*) accessions of the USDA National Small Grains Collection

**DOI:** 10.1007/s00122-022-04171-3

**Published:** 2022-07-27

**Authors:** Magdalena Ehn, Sebastian Michel, Laura Morales, Tyler Gordon, Hermann Gregor Dallinger, Hermann Buerstmayr

**Affiliations:** 1 Institute of Biotechnology in Plant Production, University of Natural Resources and Life Sciences, Konrad-Lorenz-Strasse 20, 3430 Tulln, Austria; 2 Small Grains and Potato Germplasm Research Unit, USDA-ARS, 1691 S. 2700 W., Aberdeen, ID 83210 USA

**Keywords:** *Tilletia caries*, *Triticum aestivum*, Genome-wide association mapping, Resistance breeding, Diversity panel

## Abstract

**Key message:**

Association mapping and phenotypic analysis of a diversity panel of 238 bread wheat accessions highlights differences in resistance against common vs. dwarf bunt and identifies genotypes valuable for bi-parental crosses.

**Abstract:**

Common bunt caused by *Tilletia caries* and *T. laevis* was successfully controlled by seed dressings with systemic fungicides for decades, but has become a renewed threat to wheat yield and quality in organic agriculture where such treatments are forbidden. As the most efficient way to address this problem is the use of resistant cultivars, this study aims to broaden the spectrum of resistance sources available for breeders by identifying resistance loci against common bunt in bread wheat accessions of the USDA National Small Grains Collection. We conducted three years of artificially inoculated field trials to assess common bunt infection levels in a diversity panel comprising 238 wheat accessions for which data on resistance against the closely related pathogen *Tilletia controversa* causing dwarf bunt was already available. Resistance levels against common bunt were higher compared to dwarf bunt with 99 accessions showing $\le$ 1% incidence. Genome-wide association mapping identified six markers significantly associated with common bunt incidence in regions already known to confer resistance on chromosomes 1A and 1B and novel loci on 2B and 7A. Our results show that resistance against common and dwarf bunt is not necessarily controlled by the same loci but we identified twenty accessions with high resistance against both diseases. These represent valuable new resources for research and breeding programs since several bunt races have already been reported to overcome known resistance genes.

**Supplementary Information:**

The online version contains supplementary material available at 10.1007/s00122-022-04171-3.

## Introduction

More than 100 years ago, at the beginning of the twentieth century, common bunt was a common a disease in wheat growing areas all around the world as its name suggests. In regions like the Pacific North West in the US, wheat fields were so heavily infected that the average number of spores in a spore-trap at Pullman, WA, was 36.111 per square inch in 1916, which equals almost 600 spores per gram of soil. The region therefore became known as the smut capital of the world (Bruehl 1990) since common bunt is also called stinking smut - a name hinting at the production of trimethylamines resulting in a fishy smell already at very low contamination levels (Laroche et al. 2000). In consequence, a lot of effort was put into research on the causal agents of the disease, the two closely related fungi *Tilletia caries* (D.C.) Tul. & C. Tul. (also called *Tilletia tritici* (Bjerk.) G. Winter) and *T. laevis* J.G. Kühn (also called *T. foetida* (Wallr.) Liro) and on measures to prevent them from infecting wheat plants (Bruehl 1990). The development of seed treatments with hexachlorobenzenes (HCB) during the 1950s finally provided farmers with an efficient and reliable tool to keep bunt infections at stake (Line 1993). During the following decades, research activities ceased quickly and common bunt was largely neglected until the onset of the twenty-first century and the increasing popularity of organic farming. This is reflected in the number of publications on all aspects of common bunt which were as low as 18 from 1960 to 1990 and then suddenly rose to 249 from 1991 until the end of 2020 (www.scopus.com). The concept of organic farming had already been known for a long time, but it was not until the last two decades of the twentieth century that a considerable number of farmers started adopting these practices (Kuepper 2010). As the research at hand is a collaboration between groups in the U.S. and Austria, these two countries shall serve as examples for the current significance of organic agriculture on two different continents. In the United States, 2.33 million hectares were farmed organically in 2019, leading to rank four in the list of countries with the largest organic farming areas. However, Austria as a very small country took rank 14 on this list, with 669.921 hectares (FiBL survey 2021). This makes Austria the country with the second highest percentage ( 26.1%) of organically managed farming area relative to the total arable land (FiBL survey 2021), while this value was only 0.6% in the U.S. in 2019 (Meier et al. 2021). The importance of wheat breeding for organic agriculture is emphasized by the fact that cereals are the key arable crop for organic production in both North America (The World of Organic Agriculture 2021) and Europe (Willer et al. 2021).

Treatments against common bunt infection are available for organically managed farms, but they are in most cases not as easy in their application as seed dressings for conventional farming and only provide limited control (Borgen and Davanlou 2001). Voit et al. (2012) reported that in years with high disease pressure, organic treatments only showed 65% efficiency on farms in Germany and Austria. In consequence, resistant wheat varieties can be considered the most economically efficient and environmentally friendly way of disease prevention (Borgen and Davanlou 2001; Voit et al. 2012; Matanguihan et al. 2011). A range of genes (*Bt*-genes) conferring resistance to common and/or dwarf bunt (*Tilletia controversa* J.G. Kühn) via gene-for-gene interaction have been identified in wheat (Goates 2012; Goates and Bockelman 2012; Steffan et al. 2017; Muellner et al. 2020). Apart from these qualitative resistances, also quantitative trait loci (QTL) with effect against one or both fungal diseases have recently been mapped (Bhatta et al. 2018; Mourad et al. 2018; Muellner et al. 2021; Singh et al. 2015; Fofana et al. 2008; Wang et al. 2009; Dumalasová et al. 2012). Considering results by Goates (2012) and Hoffman and Metzger (1976) who found that several of the bunt races examined in their experiments were able to overcome genotypes with known *Bt*-genes and also recent reports of resistance breakdowns against certain bunt isolates (e.g. Gladysz et al. 2021; Dumalasová 2021; Orgeur et al. 2021), the urge of identifying new resistance sources and possibly also combining several loci in a single cultivar becomes evident. One way of searching for novel resistance alleles that has become possible with the availability of high-density molecular markers for wheat is to conduct a genome-wide association study (GWAS). Successful applications of this technique have already identified SNP-markers significantly associated with bunt resistance on chromosomes 2B, 7A (Schmidt et al. 2021), 6DS (Gordon et al. 2020), 2A, 3D and 4A (Bhatta et al. 2018). A study by Mourad et al. (2018) identified more than 120 SNPs significantly associated with common bunt resistance of which SNPs on chromosomes 1A, 1B, 4A, 5B and 6A showed the highest $R^{2}$ values (between 5% and 9%).

All these studies were focused on common bunt, except for Gordon et al. (2020) who examined a diversity panel with 292 bread wheat accessions of the USDA National Small Grains Collection (NSGC) for resistance against dwarf bunt. Caused by *Tilletia controversa* J.G. Kühn, dwarf bunt is closely related to the *Tilletia* species causing common bunt. Several publications have stated that resistance against both these diseases is controlled by the same genes (Metzger and Hoffman 1978; Goates 1996, 2012), but recent findings support the hypothesis that resistance to common bunt does not automatically confer resistance to dwarf bunt and vice versa (Muellner et al. 2021). To shed more light on this question, we aim to identify marker-trait associations for common bunt resistance in the same diversity panel that was used by Gordon et al. (2020) and compare the results. Furthermore, we want to determine whether the NSGC comprises accessions which have the potential to broaden the genetic resources for common bunt resistance that can be exploited for resistance breeding in bread wheat.

## Methods

In order to test the postulated hypotheses, we evaluated a panel of 292 bread wheat accessions from the USDA NSGC for common bunt resistance. The panel is described in detail in Gordon et al. (2020). Common bunt infection data from field trials in Austria was combined with data on dwarf bunt infection levels from field trials in Utah, U.S. and genome-wide marker data generated with a 90K SNP-chip (Wang et al. 2014). Information on both phenotypic data on dwarf bunt infection and genotyping is also available in Gordon et al. (2020).

### Field trials

The NSGC panel was phenotypically evaluated in three subsequent years at the experimental station of IFA Tulln (48°19’05”N, 16°04’10”E, elevation 177 m above sea level). Sowing took place in autumn and all seed samples were artificially inoculated prior to sowing. Teliospores were harvested from infected wheat ears in previous field trials from a variety of moderately susceptible genotypes showing typical common bunt symptoms and stored at room temperature under dry conditions. The original inoculum for bunt testing at IFA Tulln consisted of a mixture of spores collected at three different locations in eastern and western Austria which represents the common bunt race spectrum in this region. Following a protocol adapted from Goates (1996) and Muellner et al. (2020) grain samples were artificially inoculated with a suspension of teliospores in a solution of methylcellulose in water (2 g of methylcellulose in 1000 ml of water). For 10 g of seeds, 0.09 g of spores were used (= 0.3 ml of spore suspension) which were added to the grain samples with a multi-dispense pipette and distributed onto the seeds by shaking.

For the trial in 2019, seeds for all genotypes were received from Tyler Gordon and sown in double-row plots of 65 cm in length in a non-replicated field trial with 17 cm spacing between rows, 33 cm spacing between plots and 50 cm spacing to the next row of plots. To facilitate sowing of further field trials, seeds were multiplied in a separate trial. Field experiments for 2020 and 2021 were sown as randomized complete block designs in two replications with single-row plots of 160 cm accompanied by a support row of equal length. This support row consisted of different short, sturdy cultivars (‘Balaton’, ‘Balitus’) and was intended to stabilize lodging-prone accessions. Spacings between rows and plots were the same as in 2019. In each year, 5 g of seeds were used per plot. Growth regulators were applied in 2020 and 2021 to prevent extensive lodging because scoring of lodged accessions becomes more complicated and error-prone.

Heading date was scored when 50% of all tillers had reached BBCH 55 (half of inflorescence emerged from flag leaf) as days after May 1. Plant height was measured as the average height per plot in cm excluding awns.

Common bunt incidence was determined in 75 randomly chosen spikes per row by cutting each spike with scissors and checking for bunted kernels. Spikes were considered infected if at least one bunt sorus was detected and incidence was calculated as the percentage of diseased spikes out of 75 spikes. Incidence was normalized (common bunt normalized incidence, CB–NI) to a range between zero and the average of susceptible cultivar ’Capo’ which we assessed in two plots as 100%.

### Molecular marker data

The final panel used for genotypic analysis in the publication at hand contained 238 accessions, after removing duplicated entries as in Gordon et al. (2020) and genotypes without phenotypic information from the Austrian field trials. All SNPs with $\le$ 5% minor allele frequency (MAF) in this reduced panel were excluded and missing values were imputed as zero. The final set of markers contained 18953 SNPs.

### Statistical analysis

All statistical analyses were carried out using R (R Core Team 2021). Best Linear Unbiased Estimates (BLUEs) were calculated for each trait observed in the replicated field trials of 2020 and 2021 using a linear mixed model of the form

1 $$\begin{aligned} P_{ik} = \mu + G_{i} + R_{k} + e_{ik} \end{aligned}$$

with $P_{ik}$ denoting the observed phenotypic value for the respective trait, $\mu$ being the grand mean, $G_{i}$ representing the effect of the $i^{th}$ genotype, $R_{k}$ being the effect of the $k^{th}$ replication and $e_{ik}$ denoting the error term. For analysis across all three environments, this model was extended to

2 $$\begin{aligned} P_{ijk} = \mu + G_{i} + E_{j} + E_{j}(R_{k}) + GE_{ij} + e_{ijk} \end{aligned}$$

to calculate BLUEs which also take the effect of the $j^{th}$ year $E_{j}$, the nested effect of replication *k* in year *j* ($E_{j}(R_{k})$) and the genotype-environment-interaction $GE_{ij}$ into account. In both models (Eqs. 1, 2) the grand mean and the genotype effect were treated as fixed effects while all other effects were modelled as random. Based on across-year BLUEs, mean values and standard errors for all phenotypic traits observed in field trials in Tulln, Austria, as well as for dwarf bunt normalized incidence were calculated for each subpopulation identified in the data by Gordon et al. (2020).

Variance components were determined using the same linear mixed model as described in Eq. 2 but only the grand mean ($\mu$) was treated as a fixed effect and all other effects were modelled as random. All models were fit with the remlf90 function from package breedR (Munoz and Sanchez 2020).

Broad-sense heritability was calculated as

3 $$\begin{aligned} H^{2} = \frac{\sigma ^{2}_{G}}{\sigma ^{2}_{G} + \frac{\sigma ^{2}_{G \times E}}{n_{E}} +\frac{\sigma ^{2}_{e}}{n_{R} \cdot n_{E}} } \end{aligned}$$

with $\sigma ^{2}_{G}$ being the genotypic variance, $\sigma ^{2}_{G \times E}$ denoting the genotype-environment-interaction, $\sigma ^{2}_{e}$ as the residual variance, $n_{R}$ as the number of replications in each year and $n_{E}$ denoting the number of test environments (Schmidt et al. 2019; Hallauer and Miranda 1986).

A principal component analysis of the genotypic data of all 238 lines was conducted using the prcomp function from the stats-package in R (R Core Team 2021) to investigate population structure.

### Genome-wide association analysis

Genome-wide linkage disequilibrium (LD) for the markers and population structure in the panel are described in Gordon et al. (2020). For detection of marker-trait associations with dwarf bunt incidence, a mixed linear model controlling for familial relationships with a kinship covariance matrix and for population stratification with two principal components (Yu et al. 2006) was the best performing model (Gordon et al. 2020). However, such a model did not perform equally well when trying to find marker-trait associations for common bunt incidence according to QQ-plots (Online Resource 10b ). We therefore applied compression of the kinship covariance matrix and determined marker-trait associations using a compressed mixed linear model [CMLM, (Zhang et al. 2010)].

Compression was achieved through partitioning around medoids clustering (Kaufman and Rousseeuw 1990) of the SNP marker data using the pamk function in R package fpc (Hennig 2020). Cluster solutions for two to 238 clusters were obtained and the optimum compression level was determined for each data set separately by fitting mixed linear models with CB–NI as the response variable, the grand mean as a fixed effect and the cluster-assignment of each genotype. Allele calls for all 18,953 markers were averaged across all genotypes assigned to a single cluster and this averaged marker profile was then assigned to each genotype in the respective cluster so that they became identical in terms of their allele calls. A similar approach has been suggested for analysis of pooled DNA of family bulks in the context of applied plant breeding programs by Michel et al. (2021) and is also described by Baller et al. (2020) for genomic predictions on pooled DNA in animal breeding. The additive relationship matrix *K* was calculated based on the averaged, clustered marker data for all 238 accessions with the A.mat function from the rrBLUP package (Endelman 2011).

Models were fitted with the mmer function of the R package sommer (Covarrubias-Pazaran 2016) and the Bayesian information criterion (BIC) was used to choose the most suitable model. For each data set (2019 to 2021 and BLUEs across years), a marker matrix as genotypic input for the final GWAS-model was prepared according to the optimal clustering solution (i.e. compression level). Genome-wide marker-trait associations were estimated using the sommer package. Mixed models with CB–NI as the response, SNP as fixed effect and genotype as a random effect, with variance-covariance specified by the *K* matrix were fitted and variance components were estimated with the *P3D* method described in Zhang et al. (2010). *P*-values, SNP effect estimates and $R^{2}$ values for each SNP in each data set were extracted from the GWA-models and multiple test correction was applied on the *p*-values using the *q*value package (Storey et al. 2020). Significant marker-trait associations were identified using a false discovery rate (FDR) of $\alpha = 0.05$.

To identify marker-trait associations for plant height and heading date, the same type of model was used as described for common bunt but the *K* matrix was calculated based on the original, non-clustered marker data.

### Evaluation of panel composition

The composition of the experimental population was initially based on data on dwarf bunt infection levels of individual accessions available in the GRIN database (https://www.ars-grin.gov/) and optimized to comprise approximately 50% dwarf bunt resistant and susceptible accessions, respectively (Gordon et al. 2020). It has been shown that races of dwarf bunt and common bunt exhibit different virulence patterns against various bunt resistance sources (Goates and Bockelman 2012; Muellner et al. 2021) and therefore, a panel optimized for dwarf bunt reactions cannot be expected to also provide optimal conditions for common bunt association mapping. To investigate how the ratio of susceptible vs. resistant accessions in an experimental population influences GWA results, we conducted a leave-one-out cross-validation based on the classification of accessions into subpopulations described in Gordon et al. (2020). Of the six subpopulations, one at a time was excluded and the GWA-procedure was repeated as described above with accessions belonging to the other five subpopulations. Since some subpopulations were composed of almost exclusively highly resistant accessions, the ratio of susceptible vs. resistant genotypes in the reduced panel changed when individual subpopulations were excluded. For this cross-validation, a non-compressed kinship matrix and the original genotypic data were used.

**Table 1:** Classification of bread wheat accessions by their country of origin into resistance classes based on data across three subsequent years

Accession origin	HR^a^	*R*^b^	*S*^c^
Azerbaijan	0	0	3
Germany	0	0	1
Iran	6	4	13
Montenegro	3	0	1
Russia	0	0	2
Serbia	9	2	7
Sweden	0	1	0
Turkey	21	8	29
USA	86	19	23
Total	125	34	79

^a^Highly resistant, $\le$ 1% CB infection

^b^Resistant, > 1% and $\le$ 10% CB infection

^c^Susceptible, > 10% CB infection

## Results

### Field trials

**Fig. 1:**
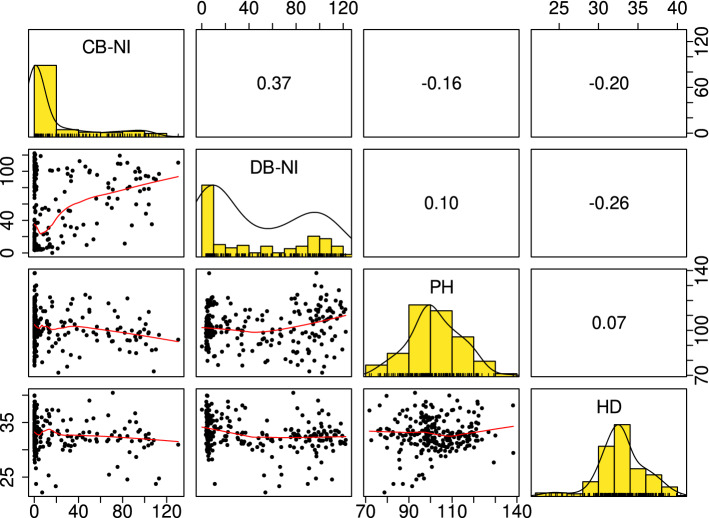
Pearson correlation coefficients, histograms and scatterplots between across-year best linear unbiased estimates (BLUEs) for normalized incidences of dwarf bunt (DB-NI) and common bunt (CB–NI) as well as plant height (PH) and heading date (HD) in the common bunt trials across all years (2019–2021) (Gordon et al. 2020)

In the whole panel 66.8% of the lines were resistant to common bunt infection with $\le$ 10% CB–NI BLUE (Table 1). Two out of three trials were replicated and Pearson correlation coefficients between replications were $r = 0.90$ for 2020 and $r = 0.60$ for 2021, both significant at $p < 0.0001$. Mean CB–NI was significantly ($\alpha = 0.05$) higher in the second replication in 2021. This could be traced back to scoring errors in the field trial and strongly deviating data points were excluded from the analysis. After this correction which concerned 18 out of 303 genotypes, correlation between replications in 2021 improved from $r = 0.60$ to $r = 0.88$. CB–NI was highly correlated between individual years ($r = 0.90$ to 0.94) with all estimates being significant at the $p < 0.0001$ level and showed strongly right-skewed distributions. Significant negative correlations were observed across years for CB–NI with plant height ($r = -0.16$, $p = 0.012$) and heading date ($r = -0.20$, $p = 0.002$), respectively (Fig. 1). A positive correlation of $r = 0.37$ ($p \le 0.0001$) was observed between across-year BLUEs for CB–NI assessed in Tulln, Austria and across-year BLUEs for DB-NI assessed in Logan, UT and in the GRIN database. The corresponding scatterplot (top left of the scatterplots in Fig. 1) also indicates that there are a lot of lines resistant to common bunt but highly susceptible to dwarf bunt and only very few which show a reversed pattern. Twenty lines showed $\le$ 1% incidence across years for both common (2019–2021) and dwarf bunt (2017–2019, Gordon et al. (2020)) (Table 2).

Infection levels in the bunt differential lines were inconsistent between years for *Bt8*, *Bt9*, *Bt15*, *BtP* and ’PI 173438’ possessing an unknown type of resistance (Table 4). In general, more of the known resistance sources are effective against common bunt compared to dwarf bunt as shown in columns “BLUE” and “DB-BLUE” in Table 4. Only for *Bt8*, *Bt14*, *Bt15*, *BtP* and the unknown resistance source of ’PI 173438’, CB–NI was higher than DB-NI across years.

Heritability of all observed traits across data sets was high ($\ge 0.84$) and highest for common bunt incidence ($H^{2} = 0.96$). For both plant height and CB–NI, the genotype effect explained the largest part of the observed phenotypic variance whereas variance in heading date was mainly explained by the environmental effect (Table 3).

**Table 2:** Accessions with high resistance levels ($\le$ 1% incidence) against both common and dwarf bunt (Gordon et al. 2020)

Accession	HD^a^	PH^b^	Status	Origin	Source/pedigree
CItr 17727	31.13	105.88	Cultivar	U.S., Idaho	From PI 178383
PI 178383	35.45	98.93	Landrace	Turkey, Hakkari	*Bt8*, *Bt9*, *Bt10*
PI 345102	36.87	120.15	Landrace	Serbia	
PI 345106	32.80	115.28	Landrace	Serbia	
PI 345428	38.13	138.19	Landrace	Montenegro	
PI 374540	33.94	103.83	Landrace	Serbia	
PI 470395	34.62	100.88	Landrace	Turkey, Hakkari	
PI 518914	39.94	108.68	Breeding line	U.S., Idaho	From PI 178383
PI 560601	32.68	88.56	Landrace	Turkey, Hakkari	
PI 560792	37.87	97.83	Landrace	Turkey, Hakkari	
PI 560842	34.06	100.64	Landrace	Turkey, Hakkari	
PI 560843	36.82	95.76	Landrace	Turkey, Hakkari	
PI 620655	32.87	91.13	Breeding line	U.S., Oregon	
PI 622967	33.24	98.44	Landrace	Iran, Esfahan	
PI 636145	38.80	97.83	Breeding line	U.S., Idaho	From PI 560603
PI 636147	38.17	96.73	Breeding line	U.S., Idaho	From PI 560603
PI 636156	38.10	92.83	Breeding line	U.S., Idaho	From PI 560795
PI 636169	36.66	96.00	Breeding line	U.S., Idaho	From PI 560843
PI 636170	36.10	88.68	Breeding line	U.S., Idaho	From PI 560843
PI 638644	31.13	102.71	Breeding line	U.S., Washington	

Values for heading date and plant height are best linear unbiased estimates (BLUEs) across trials in Tulln, Austria, from 2019–2021

^a^Heading date in days after May 1

^b^Plant height in cm

**Table 3:** Average, minimum and maximum values for individual years and BLUEs across years , variance components (rows 5–9) and broad-sense heritability estimates ($H^{2}$) for phenotypic traits observed in field trials from 2019 to 2021

	HD^a^	PH^b^	CB–NI^c^
2019	34.2 (25–42)	109.0 (55–150)	18.0 (0–131)
2020	25.39 (16–40)	100.1 (65–145)	15.57 (0–123.7)
2021	39.4 (25–48)	97.63 (55–150)	22.7 (0–158.7)
BLUE	33.08 (22.2–43.5)	102.3 (55–143.8)	18.88 (0–130.6)
$V_\mathrm {{Genotype}}$	8.9	127.1	888.0
$V_\mathrm {{Environment}}$	50.5	48.9	12.0
$V_\mathrm {{Replication}}$	0	2.7	4.5
$V_{G\times E}$	0.8	14.0	44.7
$V_\mathrm {{error}}$	1.2	113.8	118.8
$H^{2}$	0.95	0.84	0.96

^a^Heading date in days after May 1

^b^Plant height in cm

^c^Common bunt normalized incidence

### Subpopulations and disease reaction

**Fig. 2:**
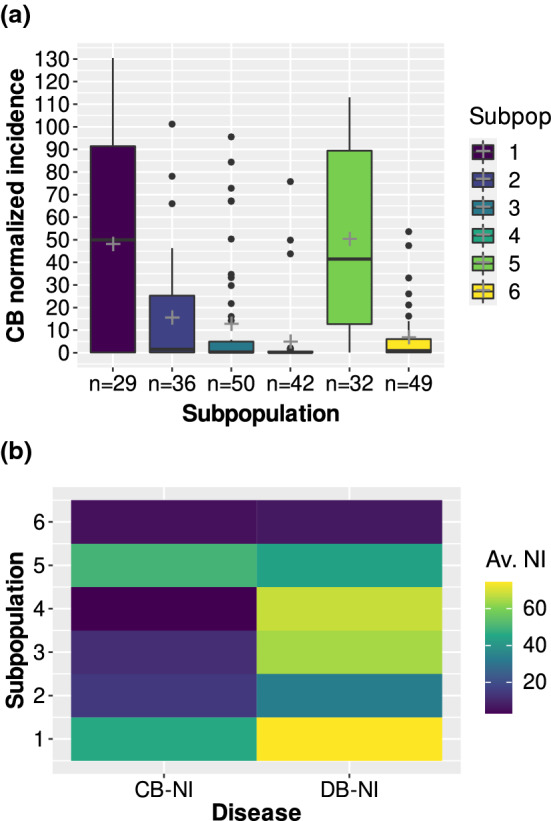
**a** Best linear unbiased estimates (BLUEs) across three years for common bunt normalized incidence (CB–NI) in percentages for genotypes assigned to different subpopulations. Number of genotypes per subpopulation is shown on the *x*-axis, crosses mark average CB–NI. **b** Heatmap comparing subpopulation averages of BLUEs across years for normalized incidence (NI) of dwarf bunt (DB-NI) and CB–NI (Gordon et al. 2020)

**Fig. 3:**
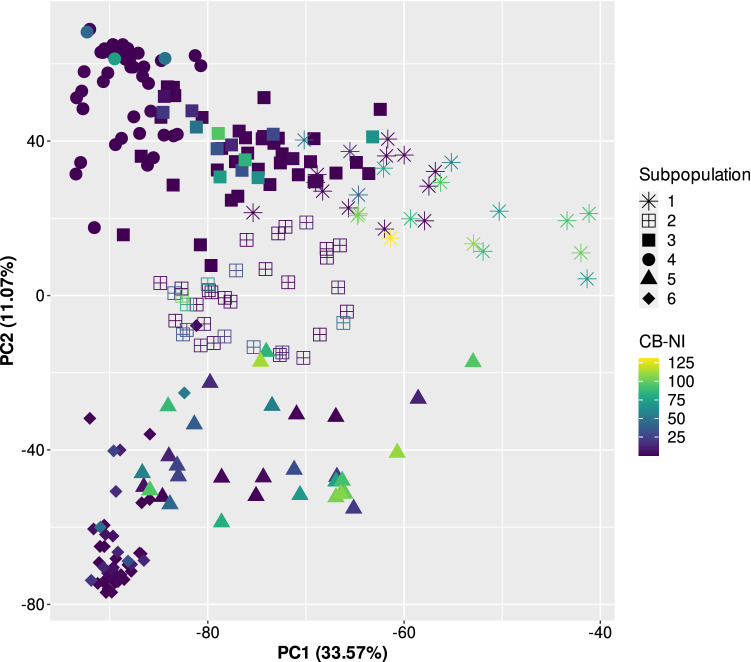
Scatterplot of the first two principal components of the 238 accessions used for association mapping. Individual subpopulations in the panel are discriminated by shapes of the data points. Colours of individual data points indicate across-year best linear unbiased estimates (BLUEs) of normalized common bunt incidence (CB–NI) levels of the respective genotypes (Gordon et al. 2020)

Accessions from Iran, Serbia and Turkey showed the highest proportions of susceptible lines whereas accessions originating from the U.S. were for the most part highly resistant (Table 1). Individual subpopulations reacted differently to common bunt compared to dwarf bunt (Fig. 2b). While genotypes assigned to subpopulations three and four showed the second and third highest dwarf bunt infection levels, they had low average CB–NI (Online Resource 1). Subpopulations two and six showed similar reactions to both diseases. High variation in CB–NI was observed for subpopulations one and five (Figs. 2a and 3). Average infection levels for common bunt were lower than for dwarf bunt in all subpopulations except subpopulation five. Please note that only dwarf bunt data on those genotypes for which CB–NI could be assessed in all three years was used for the analysis, resulting in 238 accessions compared to 246 used by Gordon et al. (2020). While accessions in subpopulations two, four and six, respectively, clustered together and showed low variation in CB–NI in a PCA heatmap (Fig. 3), genotypes belonging to subpopulations one, three and five, respectively, were more scattered across the principal component plot. Variation in plant height also differed between subpopulations with highest variation in subpopulation three and a comparably narrow range of observed heights in subpopulation six. Such patterns were not found for heading date. Variation in heading date was similar and standard errors were low across all subpopulations (Online Resource 1).

### Marker-trait associations

Based on model fit in terms of BIC for models with compressed kinship matrices, we chose the best fitting model and thereby the ideal number of clusters, i.e. groups, for each data set (2019–2021 and BLUEs across years). While for 2020 data, the ideal number of clusters was approximately the same as the number of genotypes (236), higher compression was optimal for 2021 (155 clusters). The ideal number of clusters for 2019 was 176 and across years, the optimum compression was reached with 230 clusters (Online Resource 8).

**Fig. 4:**
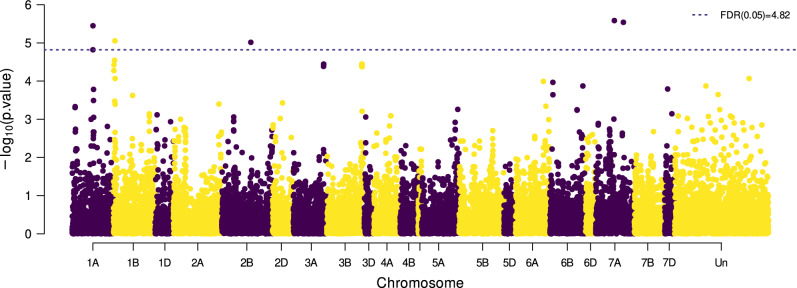
Manhattan plot showing marker-trait associations for best linear unbiased estimates (BLUEs) of normalized common bunt incidence across all three years (2019–2021). The dashed line marks a significance threshold of $\alpha = 0.05$

With a model that corrected for relatedness using a compressed kinship matrix with the optimum compression level for each year, six SNP markers were found to be significantly (FDR-adjusted *p*-value $\le 0.05$) associated with CB–NI in at least one out of four data sets (Fig. 4) and thereof, four SNPs (in the following called *CB-1A*, *CB-2B*, *CB-7A1* and *CB-7A2*) showed significant associations in two of the data sets (Table 5). The resistance conferring allele was the prevalent one for all four SNPs in the panel under investigation and allele frequencies ranged from 91.2% to 94.1%. Differences in average CB–NI levels between accessions carrying the resistant vs. the susceptible allele ranged from 29.4% for *CB-7A1* to 52.1% for *CB-2B*. In the data set for 2021, no markers showed significant associations with CB–NI, but p-values indicated that SNPs *CB-1A* and *CB-2B* which were significantly associated with resistance in other years also might play a role in bunt resistance in 2021 (Online Resource 9).

Allele calls at the four SNP positions associated with CB–NI in more than one data set for all lines in the bunt differential set for which both genotypic and phenotypic data was available reflect the high allele frequencies of the resistance conferring alleles. Those three differential lines showing the highest CB–NI and DB-NI levels (*Bt0*, *Bt2* and *Bt7*) are the only genotypes which lack two of the resistance conferring alleles, all other lines in the differential set have the resistant allele in at least three out of four SNP positions (Online Resource 2).

Association mapping for heading date identified two markers on chromosome 7B in an interval of 9.753 to 9.754 Mbp and one marker on chromosome 7D at 72.95 Mbp to be significantly (FDR-adjusted *p*-value $\le 0.05$) associated with time to heading in at least two out of four data sets (Online Resource 4). No significant marker-trait associations were detected for plant height.

**Table 4:** Phenotypic scores for dwarf bunt (DB) and common bunt (CB) normalized incidence for the bunt differential set and the susceptible cultivar ’Capo’ used for normalization

Accession	Name	*Bt*-gene	DB-BLUE^a^	CB 2019	CB 2020	CB 2021	CB BLUE^b^
.	Capo	Susceptible	.	100	100	100	100
PI 209794	Heines VII	Susceptible	111.2	82.7	78.0	100.8	86.5
PI 554101	Selection 2092	*Bt1*	104.0	0	0	0	0.1
PI 554097	Selection 1102	*Bt2*	119.2	84.7	56.8	93.1	77.5
CItr 6730	Ridit	*Bt3*	51.2	0	0	2.1	0.9
PI 11610	CI 1558	*Bt4*	120.4	0	1.7	0	0.8
CItr 11458	Hohenheimer	*Bt5*	32.1	0	2.5	0	4.0
CItr 10061	Rio	*Bt6*	67.4	0	0	0	0.1
PI 554100	Selection 50077	*Bt7*	112.7	44.6	48.7	.	49.9
PI 554120	M72-1250	*Bt8*	7.5	0	1.7	33.9	13.6
PI 554099	R63-6968	*Bt9*	55.3	13.4	3.4	13.8	9.9
PI 554118	R63-6982	*Bt10*	37.0	0	2.5	5.3	3.1
PI 554119	M82-2123	*Bt11*	4.5	0	0.8	0	1.0
PI 119333	1696	*Bt12*	3.4	0	0	0	0.6
PI 181463	Thule III	*Bt13*	11.0	0	2.7	1.1	1.6
CItr 13711	Doubbi	*Bt14*	3.7	0	6.6	3.2	3.9
CItr 12064	Carleton	*Bt15*	14.8	37.4	6.2	21.2	19.6
PI 173437	7838	*BtP*	0.1	18.6	4.4	29.6	16.2
PI 173438	7845	Unknown	0.1	0	16.1	19.0	13.5
PI 178383	6256	*Bt8,9,10*	4.5	0	0	0	0.1
PI 476212	SM Selection 4	Unknown	4.0	0	0	2.1	0.9

^a^Normalized dwarf bunt incidence across four data sets derived from Gordon et al. (2020)

^b^Normalized common bunt incidence across three data sets (2019–2021)

**Table 5:** SNP markers significantly (FDR-adjusted *p*-value $\le 0.05$) associated with normalized common bunt incidence in data from individual field trials in Tulln, Austria, from 2019 to 2021 or best linear unbiased estimates (BLUEs) across all three trials

SNP	Chromosome	bp^a^	Data set	AF^b^	*R*_CB–NI_c	*S*_CB–NI_^d^	LOD^e^	${r^{2}}$
RAC875_c31133_464	1A	473.965.765	BLUEs	91.6	16.2	51.5	4.82	0.08
RAC875_c31133_77	1A	473.966.540	2020, BLUEs	91.2	15.9	53.2	5.18, 5.44	0.08, 0.09
BS00032266_51	1B	11.181.473	BLUEs	92.9	17.0	58.8	5.05	0.08
Ku_c71357_859	2B	581.704.044	2019, BLUEs	94.1	16.4	68.5	5.80, 5.01	0.09, 0.08
Ku_c5529_824	7A	335.991.471	2020, BLUEs	93.3	17.1	46.5	5.61, 5.58	0.09
RAC875_c23665_68	7A	629.801.516	2020, BLUEs	91.2	15.7	57.5	5.46, 5.53	0.08, 0.09

^a^Position in bp

^b^Allele frequency of the resistant allele in %

^c^Average CB–NI score (based on BLUEs) for accessions carrying the resistant allele

^d^Average CB–NI score (based on BLUEs) for accessions carrying the susceptible allele

^e^FDR-adjusted $-log_{10}(p)$-value

## Discussion

The inoculation method to provoke common bunt infections used at IFA Tulln was proven to be effective over several years of field trials and led to successful infestation of experimental genotypes with common bunt in all years (2019–2021). The susceptible cultivar ’Capo’ and the susceptible control line in the bunt differential set, ’Heines VII’ showed high infection levels in all field trials (Table 4). The comparably low correlation between replications in 2021 resulting from scoring errors that had to be corrected remains unexplained. Methods and procedures used in 2021 were no different from previous years which showed good correlations between replications and no plausible causes for the observed discrepancy could be identified.

Of the genotypes tested in this study, 42% (99 out of 238 lines) were highly resistant ($\le$ 1% CB–NI) to common bunt in each data set. Compared to 11.38% of consistently highly dwarf bunt resistant lines (Gordon et al. 2020), this ratio is very high. Accessions were originally chosen to be 50% dwarf bunt resistant and susceptible, respectively, and to represent many different geographic origins. An ideal situation would be to have an approximately equal number of resistant and susceptible accessions from each geographic region, but this was already not the case for dwarf bunt. The six subpopulations identified in the diversity panel showed variation in their mean DB-NI levels as described in Gordon et al. (2020) and the same problem occurred for CB–NI levels (Fig. 3, Online Resource 1). Contrary to DB-NI, though, the majority of all subpopulations showed very low CB–NI below the overall average of 19.4% (values based on BLUEs across years) while only two subpopulations had higher than average CB–NI. As variation for CB–NI was low in the panel and alleles that confer susceptibility were rare, fitting a standard kinship matrix lead to overfitting, leaving no variation to be explained by putative QTL. We tackled this problem by using compressed kinship matrices as described in Zhang et al. (2010), reducing matrix complexity and facilitating association of observed variation with genetic loci. Compressing kinship matrices has been shown to improve model fit and increase statistical power compared to general linear models (GLM) and non-compressed mixed linear models (MLM) if the optimum number of groups for clustering is chosen as compression level (Zhang et al. 2010).

In view of these challenges and in line with the suggestions by Gordon et al. (2020), we would therefore recommend to take extra care when assembling a diversity panel intended for GWA analysis in order to ensure approximately equal percentages of resistant and susceptible accessions in the overall panel as well as for individual regions of origin. The benefits of a balanced data set with approximately equal variation for a certain trait in each subpopulation also become visible when considering the other traits assessed in the common bunt trials in Tulln. While variation in plant height also differed between subpopulations, variation in heading date was more evenly distributed. With a mixed model correcting for familial relationships with a standard kinship matrix, QQ-plots indicated appropriate modelling of the data and significant marker-trait associations were detected (Online Resources 4 and 10c). For plant height, on the other hand, similar problems as for CB–NI were encountered and would have to be addressed in a separate analysis.

Subpopulation six was the only one exhibiting consistently low incidence levels for both DB-NI and CB–NI. It is mainly composed of landraces from Hakkari province in Turkey and U.S. breeding lines that incorporate such landraces in their pedigrees as described in Gordon et al. (2020). The province is located in a mountainous region characterized by a continental climate with snowy winters and dry summers matching the Köppen-Geiger climate classifications of *Dsa* to *Dsc* (Beck et al. 2018; Turkish State Meteorological Service 2022). Such climatic conditions are especially favourable for dwarf bunt infections and have lead to the evolution of highly dwarf bunt resistant landraces in this region (Bonman et al. 2006). Common bunt needs less specific conditions to infect its host and the occurrence of resistant genetic resources is not limited to narrow geographic regions as shown in the study by Bonman et al. (2006). We therefore hypothesize that genotypes from Hakkari province might be of special interest to breeders and scientists searching for high levels of resistance against both types of bunt diseases.

CB–NI showed high heritability ($H^{2} = 0.96$) which is comparable to previous studies using data from artificially inoculated field trials (Muellner et al. 2020, 2021; Chen et al. 2016; Wang et al. 2019). Correlations between individual years were also high, but no SNP was found to be significantly associated with CB–NI in more than two of the four data sets. There are only few studies dealing with GWA for common bunt so far (Mourad et al. 2018; Bhatta et al. 2019). To our knowledge, the only one providing results for multiple years is the one by Gordon et al. (2020), working with the same panel as the study at hand but investigating dwarf bunt resistance on the accessions. They observed a similar pattern of differing results in marker-trait associations across years which could possibly be caused by factors like marker-by-environment interactions or the application of the stringent FDR-threshold of $\alpha = 0.05$.

Four markers were significantly associated with CB–NI in two data sets out of which two markers on chromosomes 1A and 7A, respectively, overlap with or are in proximity of regions previously reported to be associated with bunt resistance. Marker *CB-1A* is located at 473.97 Mbp on chromosome 1A. In addition, marker *RAC875_c31133_464* which is only 775 bp away from *CB-1A* was also found to be significantly associated with common bunt resistance in BLUEs across years. Muellner et al. (2020) have mapped a locus conferring dwarf bunt resistance to this chromosomal region between 380.97 and 516.67 Mbp while Chen et al. (2016) mapped a dwarf bunt resistance locus to a region between 74 and 76 cM on chromosome 1A. The peak marker for this 1A locus was *Xcfa2129* in their study. Marker *IWA6553* is neighbouring *Xcfa2129* and is located at 503.31 Mbp according to the *Triticeae Toolbox* (available via https://wheat.triticeaetoolbox.org) (Blake et al. 2016). Muellner et al. (2020) also included common bunt resistance in their study and detected a QTL in close proximity (starting at 490.09 Mbp) of *CB-1A* which conferred high levels of resistance to common bunt in their mapping populations.

Two markers on different positions of chromosome 7A were found to be associated with CB–NI in this study. Wang et al. (2019) mapped dwarf bunt resistance to a region on chromosome 7A approximately 100 Mbp away from marker *CB-7A2* at 629.80 Mbp. Chromosome 7A was also identified to be associated with common bunt resistance in earlier studies. Fofana et al. (2008) mapped a QTL with a small but consistent effect against common bunt infection to a region on the long arm of chromosome 7A. The location of the second marker found on 7A in this study, *CB-7A1* located at 336.00 Mbp, is ambigous. While reported to be on 7A in the annotation data of the wheat 90K SNP chip, this marker is recorded on chromosome 7B at 339.21 Mbp in the wheat 90K Array Consensus and RefSeq v1.0 (Blake et al. 2016; IWGSC et al. 2018) and also on 7B but at 342.46 Mbp in the wheat RefSeq v2.1 (Zhu et al. 2021). This discrepancy might be a possible explanation why no association with common or dwarf bunt has been reported for this location on chromosome 7A in any study published to date.

To our knowledge, marker *CB-2B* identified to confer common bunt resistance in this study has not yet been reported in any other publication. Bhatta et al. (2018) report marker-trait associations for common bunt for two markers at 795.3 Mbp and 799.3 Mbp, respectively. Chromosome 2B has been reported to harbour bunt resistance gene *Bt1*, which has not yet been mapped or further characterized (Sears et al. 1960; McIntosh et al. 1998). *Bt1* has been shown to provide resistance against several isolates of *T. caries* and *T. laevis * (Goates 2012) as well as against prevalent isolate mixtures in Austria used in field tests at IFA Tulln (data not shown). *PI 554101*, the accession for *Bt1* in the differential set, possesses the resistant allele for all four SNPs associated with CB–NI in more than one year in our study (Online Resource 2), so further work would be required to determine if marker *CB-2B* could be linked to resistance gene *Bt1*.

Marker *BS00032266_51* on chromosome 1B (located at 11.18 Mbp) was only found to be associated with CB–NI in the BLUEs across all years and corresponds with regions identified to confer common bunt resistance by Muellner et al. (2020) (4.35 to 38.91 Mbp) and Singh et al. (2015) (peak marker at 13.0 Mbp). Fofana et al. (2008) also detected a QTL on the short arm of chromosome 1B at $\sim$ 19.3 cM in a mapping population derived from the cross $\mathrm {RL4452 \times \,'AC\,Domain'}$. While the 1B-marker only crossed the significance threshold of $FDR = 0.05$ in a single data set and showed comparably low effect sizes in our experiment, it was the most effective locus explaining the largest part of the phenotypic variance in the three studies mentioned above. In general, wheat chromosome 1B plays an important role in bunt resistance as several other authors have also reported markers or QTL associated with bunt incidence at different positions on 1B (Dumalasová et al. 2012; Wang et al. 2009; Zou et al. 2017; Bhatta et al. 2019; Mourad et al. 2018; Galaev et al. 2018). Furthermore, reports on the initial set of ten bunt differentials (Hoffman and Metzger 1976) state that three different resistance genes (*Bt4*, *Bt5* and *Bt6*) are located on chromosome 1B (Schmidt et al. 1969; McIntosh et al. 1998).

Allele frequencies for all markers passing the significance threshold in this study were very high - both in the overall population but also in individual subpopulations (Online Resource 3). It has been discussed by multiple authors (Dickson et al. 2010; Gibson 2012; Zhu et al. 2011) that the detection of rare variants acting as causal agents for the trait of interest in association mapping is difficult and comes with challenges. In our study, the rare variant is the one causing infection while the desired genotype leading to resistance is abundant. This would be unexpected in most other experimental or natural populations where bunt resistance would be caused by rare alleles. Nevertheless, the pre-selection for dwarf bunt resistance applied while assembling the diversity panel used in this study caused a strong deviation in allele frequencies of loci conferring common bunt resistance compared to what would be expected without pre-selection. By conducting a kind of leave-one-out cross-validation with exclusion of one subpopulation at a time, we investigated the influence of high percentages of highly resistant lines on the GWA results. The robustness of our results is supported, as loci found to be significantly associated with CB–NI levels in the full panel of lines also frequently passed the significance threshold of an FDR-adjusted p-value of 0.05 if one of the subpopulations was excluded. Especially exclusions of subpopulations three and four, consisting almost entirely of highly resistant genotypes (Fig. 2) and showing allele frequencies of close to or equal to 100% for the resistance conferring allele (Online Resource 3), were of interest as these diminished the number of highly resistant lines by 26 and 30, respectively - i.e. by more than a quarter of the total number. Since the reported markers were also found to be significantly associated with CB–NI in this cross-validation process (Online Resource 11 ), we conclude that our methodology was appropriate in terms of coping with the rare nature of susceptible variants and results can be regarded as robust.

Comprehensive data on both common and dwarf bunt incidence levels is now available for the diversity panel investigated in this study which consists of accessions from the USDA National Small Grains Collection. This gives both the scientific community and breeders access to genotypes with high levels of resistance to both bunt diseases. In total, 20 accessions have been identified which had a mean DB-NI of $\le$ 1% according to Gordon et al. (2020) and at the same time showed $\le$ 1% CB–NI in each of the four data sets used in our study (Table 2). These accessions originate from various geographic origins and thereby may provide valuable new genetic variation for research and breeding programs aimed at creating bunt resistant material. To validate the identified common bunt resistance loci in other wheat populations or panels and to facilitate their usage in (pre-)breeding programs, KASP markers for the respective QTL regions should be developed. This will be subject of future bunt research projects at IFA Tulln.

## Supplementary Information

Below is the link to the electronic supplementary material.

Supplementary file 1 (xlsx 4208 KB)

Supplementary file 2 (pdf 22164 KB)

## Data Availability

All data generated or analysed during this study are included in this published article and its supplementary information files. Data on common bunt phenotypes has been provided to Harold E. Bockelman for addition to the GRIN database.
