# Supplementary material for: Genome-wide association mapping identifies common bunt (Tilletia caries) resistance loci in bread wheat (Triticum aestivum) accessions of the USDA National Small Grains Collection
Source: Theor Appl Genet. 2022 Jul 27;135(9):3103–15. doi: 10.1007/s00122-022-04171-3 (PMC9668943; doi:10.1007/s00122-022-04171-3)
Supplement: Supplementary file 2 — (pdf 22164 KB) [file 122_2022_4171_MOESM2_ESM.pdf]

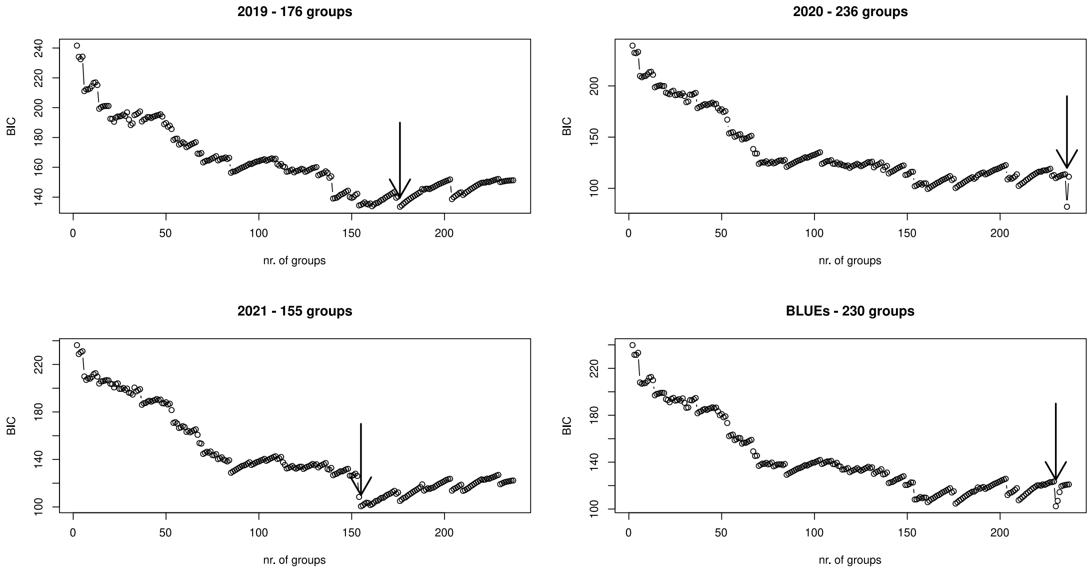

Supplementary File 8: BIC-values (y-axis) for model fit with different compression levels. Compression was applied by clustering genotypes into different numbers of groups (from two to 238, x-axis) according to their marker scores for 18953 SNP markers and thereby obtaining compressed kinship matrices. BIC values were obtained from mixed models with common bunt normalized incidence as the response variable and the compressed kinship matrices as the random effect. The optimum compression level for each data set is indicated by the lowest BIC value and marked with arrows in the plots.

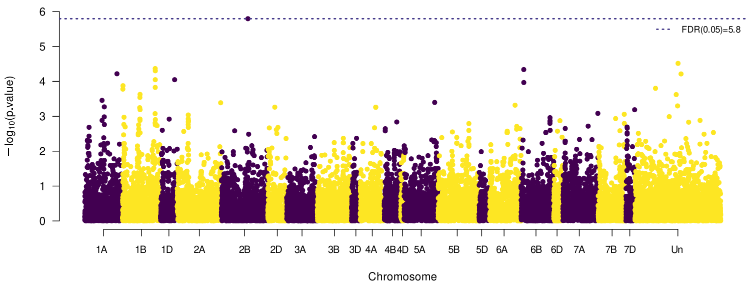

(a) 2019 - 176 groups

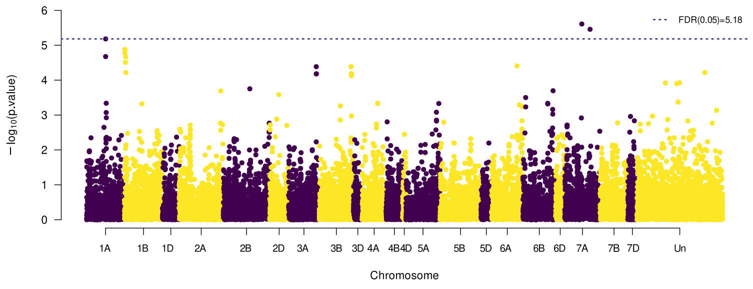

(b) 2020 - 236 groups

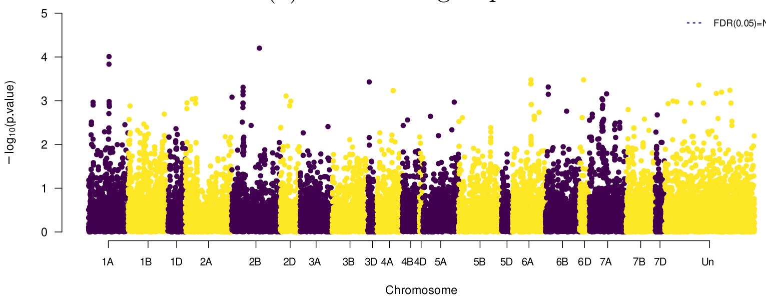

(c) 2021 - 155 groups

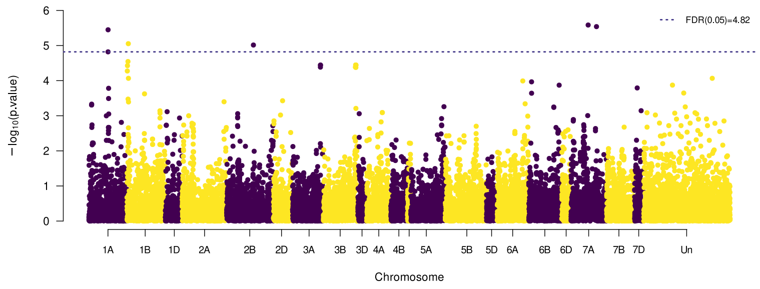

(d) BLUEs - 230 groups

Supplementary File 9: Manhattan plots for p-values obtained from mixed models including a compressed kinship matrix with an optimal compression level as a random effect for each data set. Plots are shown for individual data sets from 2019 to 2021 (a-c) and best linear unbiased estimates (BLUEs) across years (d). SNP-markers significantly (FDR-adjusted p-value ≤ 0.05) associated with CB infection are located above the dashed lines which represent FDR-adjusted thresholds of $\alpha = 0.05$.

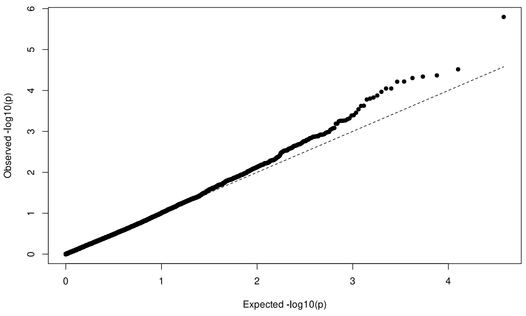

(a) 2019 - 176 groups

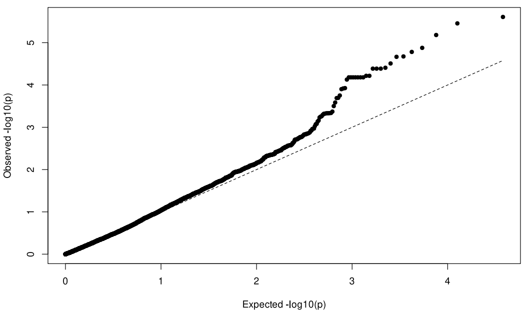

(b) 2020 - 236 groups

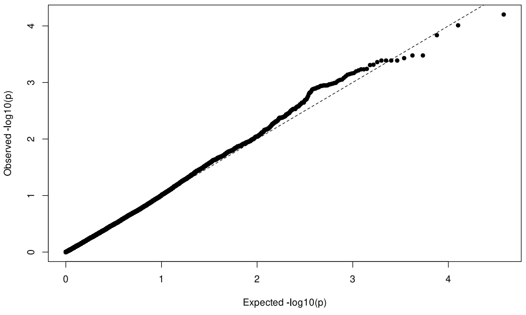

(c) 2021 - 155 groups

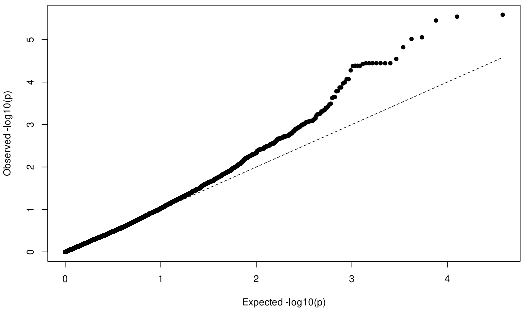

(d) BLUEs - 230 groups

Supplementary File 10a: QQ-plots for observed vs. expected $-log_{10}(p)$-values for marker-trait associations with normalized common bunt incidence from mixed models using K matrices with ideal compression levels to correct for familial relationships for each data set. Plots are shown for individual data sets from 2019 to 2021 (a-c) and best linear unbiased estimates (BLUEs) across years (d)

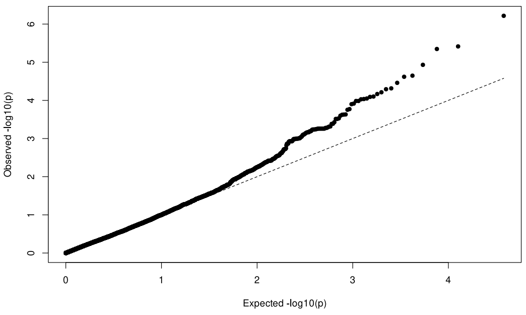

(a) 2019

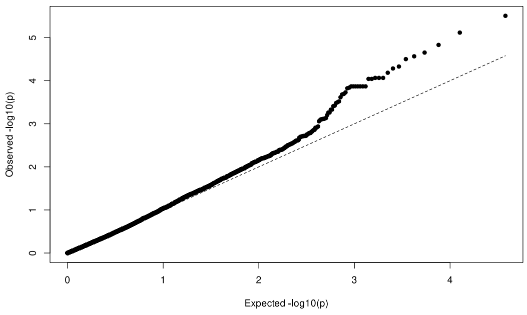

(b) 2020

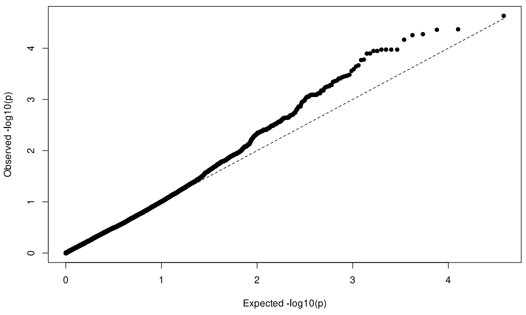

(c) 2021

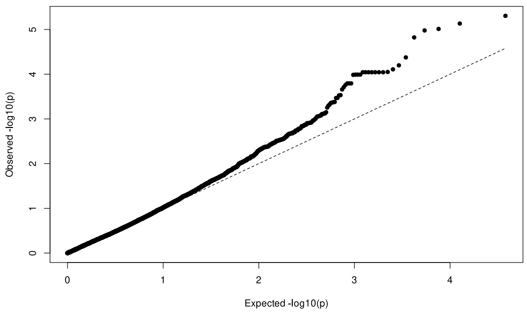

(d) BLUEs

Supplementary File 10b: QQ-plots for marker-trait associations with normalized common bunt incidence obtained from mixed models including a K matrix to correct for familial relationships as a random effect and two principal components to correct for population stratification as fixed effects for each data set. This type of model was used to analyse marker-trait associations for dwarf bunt by Gordon et al., 2020. Plots are shown for individual data sets from 2019 to 2021 (a-c) and best linear unbiased estimates (BLUEs) across years (d)

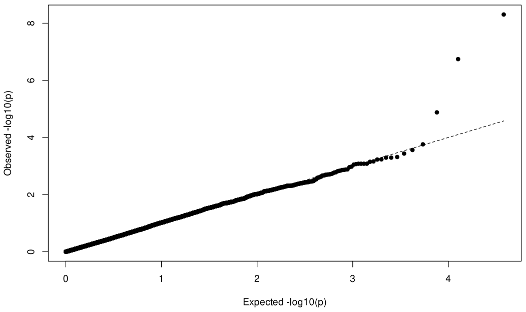

(a) 2019

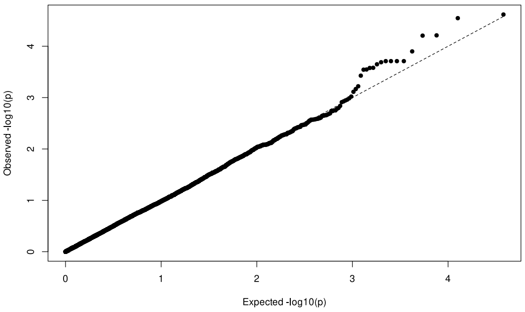

(b) 2020

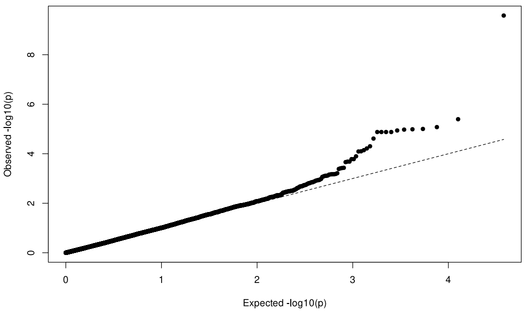

(c) 2021

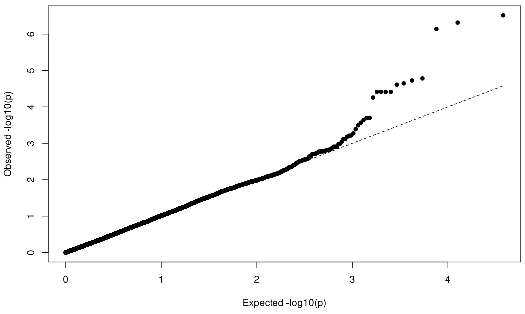

(d) BLUEs

Supplementary File 10c: QQ-plots for marker-trait association with heading date obtained from mixed models including a standard, non-compressed K matrix to correct for familial relationships as a random effect for individual years 2019 to 2021 (a-c) and best linear unbiased estimates (BLUEs) across years (d)

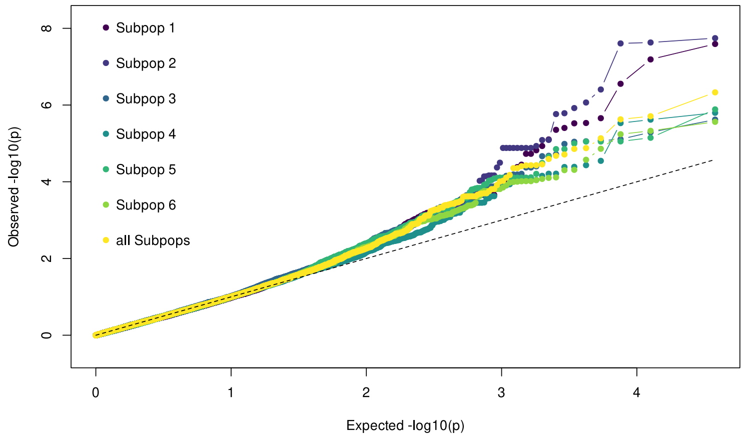

(a) 2019

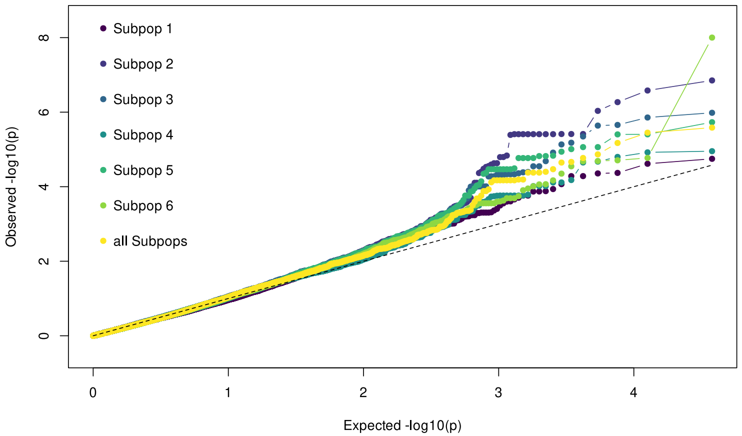

(b) 2020

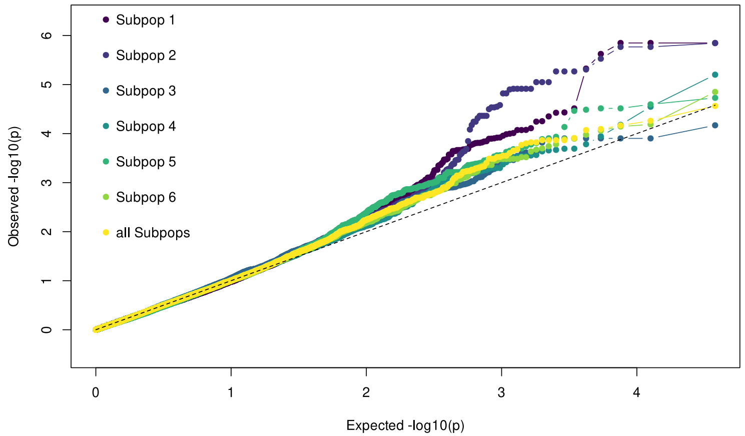

(c) 2021

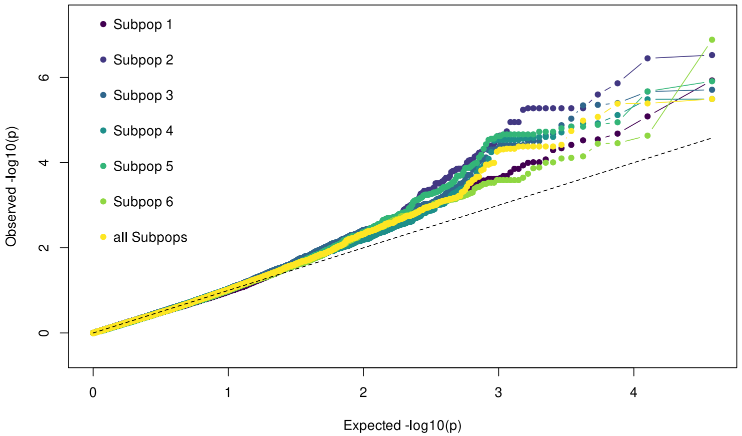

(d) BLUEs

Supplementary File 11: QQ-plots for data from single years (a-c) and across year best linear unbiased estimates (BLUEs) (d) showing expected vs. observed FDR-adjusted $-log_{10}(p)$-values for a leave-one-out cross-validation procedure excluding one out of six subpopulations at a time from the analysis, respectively (Subpop 1 to Subpop 6). This affects the ratio of susceptible vs. resistant accessions in the panel and leads to variation in GWA results

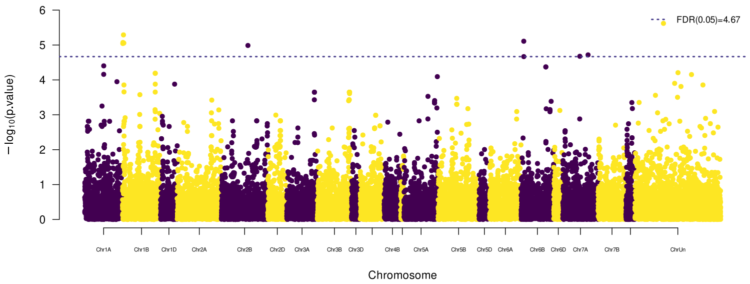

(a) 2019

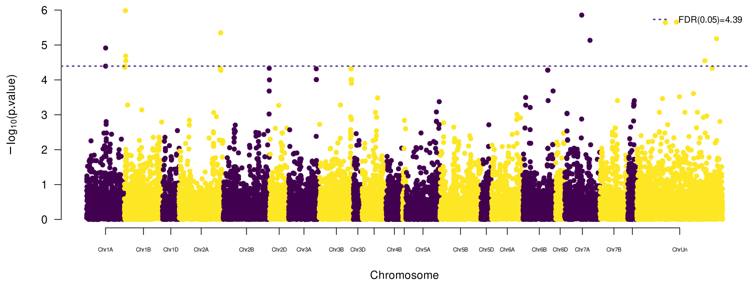

(b) 2020

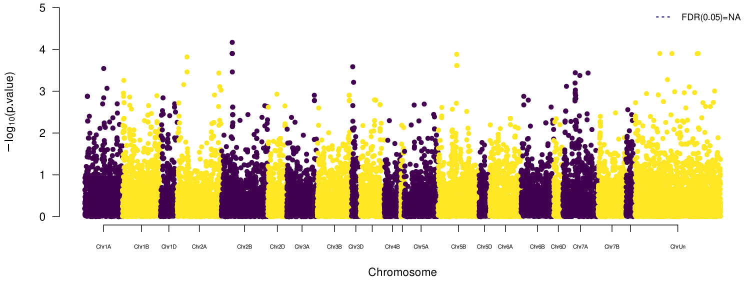

(c) 2021

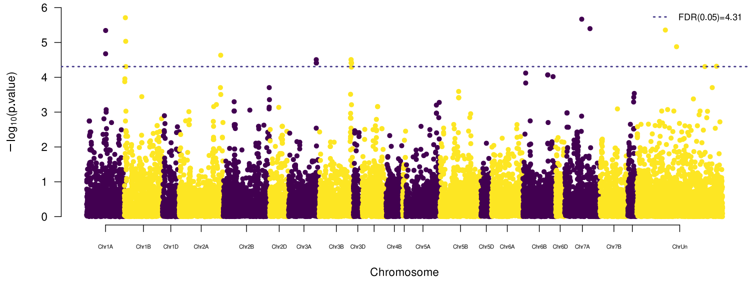

(d) BLUEs

Supplementary File 12a: Manhattan plots for p-values obtained from mixed models using a kinship matrix to correct for relatedness and excluding all accessions belonging to subpopulation three (out of six subpopulations identified in the data set by Gordon et al., 2020). This exclusion shifted the ratio of resistant vs. susceptible lines towards susceptibility compared to the full panel through reduction of the number of highly resistant lines by 26. Plots are shown for individual data sets from 2019 to 2021 (a-c) and best linear unbiased estimates (BLUEs) across years (d). SNP-markers significantly (FDR-adjusted p-value ≤ 0.05) associated with CB infection are located above the dashed lines which represent FDR-adjusted thresholds of $\alpha = 0.05$.

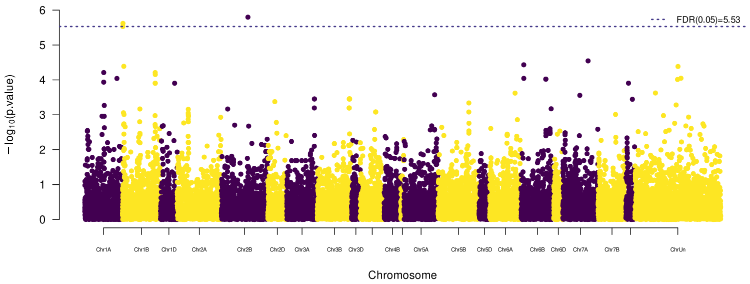

(a) 2019

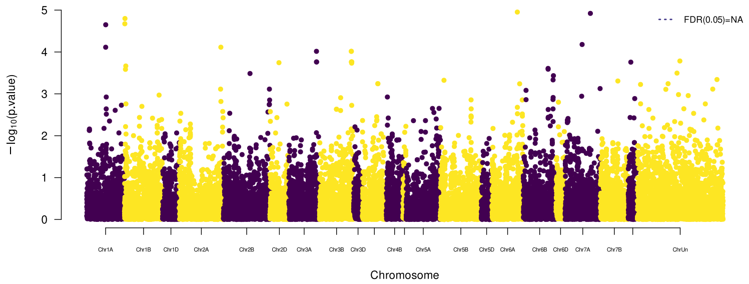

(b) 2020

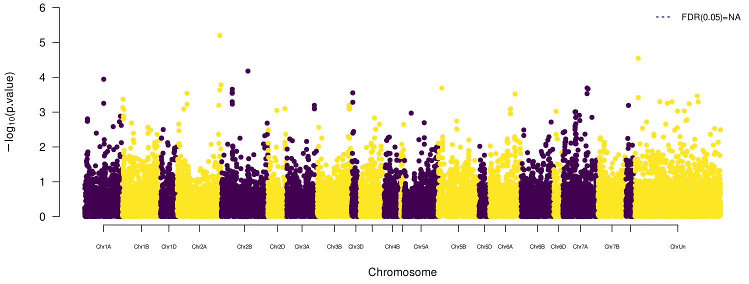

(c) 2021

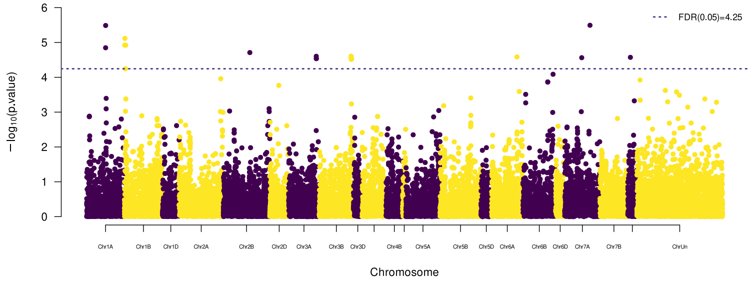

(d) BLUEs

Supplementary File 12b: Manhattan plots for p-values obtained from mixed models using a kinship matrix to correct for relatedness and excluding all accessions belonging to subpopulation four (out of six subpopulations identified in the data set by Gordon et al., 2020). This exclusion shifted the ratio of resistant vs. susceptible lines towards susceptibility compared to the full panel through reduction of the number of highly resistant lines by 30. Plots are shown for individual data sets from 2019 to 2021 (a-c) and best linear unbiased estimates (BLUEs) across years (d). SNP-markers significantly (FDR-adjusted p-value ≤ 0.05) associated with CB infection are located above the dashed lines which represent FDR-adjusted thresholds of $\alpha = 0.05$.
